# From benign to malignant: unveiling invasive squamous cell carcinoma following resection of perianal condyloma acuminatum: a case report

**DOI:** 10.1093/jscr/rjae266

**Published:** 2024-04-26

**Authors:** Kristali Ylli, Wala ElJack, Chloe Spillane, Mar Cotter, Shane Killeen

**Affiliations:** Department of Surgery, Mercy University Hospital, Grenville Place, Centre, Cork T12 WE28, Ireland; Department of Surgery, Cork University Hospital, Wilton, County Cork, T12 DC4A, Ireland; Department of Surgery, Beaumont Hospital, Beaumont Road, Beaumont, Dublin D09V2N0, Ireland; Department of Surgery, Cork University Hospital, Wilton, County Cork, T12 DC4A, Ireland; Department of Surgery, Mercy University Hospital, Grenville Place, Centre, Cork T12 WE28, Ireland

**Keywords:** condyloma acuminatum, invasive squamous cell carcinoma, perianal mass, surgical excision, IGAP flap reconstruction

## Abstract

This case illustrates the surgical management of a perianal mass, initially misdiagnosed as condyloma acuminatum in a male patient in his late 50s, later identified as invasive squamous cell carcinoma following excision. Despite extensive preoperative evaluation, the lesion's malignancy was confirmed through histopathology. The significant, fungating mass required a multidisciplinary approach, culminating in a pT3 staging and additional wide excision with inferior gluteal artery perforator flap reconstruction. This case underscores the critical importance of surgical diligence and adaptability, highlighting the role of comprehensive surgery in both diagnosis and treatment of complex perianal malignancies, and reaffirms the value of a multidisciplinary team in achieving favourable outcomes.

## Introduction

Perianal masses encompass a wide array of clinical entities, spanning benign anal pathologies such as hemorrhoids to more sinister diagnoses like anal carcinoma. The inherent diagnostic ambiguity of perianal lesions necessitates a comprehensive surgical assessment and an astute approach to management [1] . This case report delves into the surgical intricacies encountered in a late 50s male patient, initially presenting with a lesion presumed to be condyloma acuminatum—a benign proliferation frequently linked to human papillomavirus (HPV) infection [2]. Remarkably, subsequent surgical excision and detailed histopathological evaluation revealed the lesion to be invasive squamous cell carcinoma (SCC). This unexpected malignancy highlights the indispensable role of surgical intervention not only in the treatment but crucially in the accurate diagnosis of perianal masses [3, 4]. Through this case, we underscore the necessity of a wide-ranging differential diagnosis and the importance of a vigilant approach towards potential malignancies in the evaluation of perianal lesions [3]. This case report underscores the complexities and challenges intrinsic to differentiating between benign and malignant perianal pathologies, highlighting the indispensable role of surgical expertise in delivering optimal patient care [3].

## Case report

**Figure 1:**
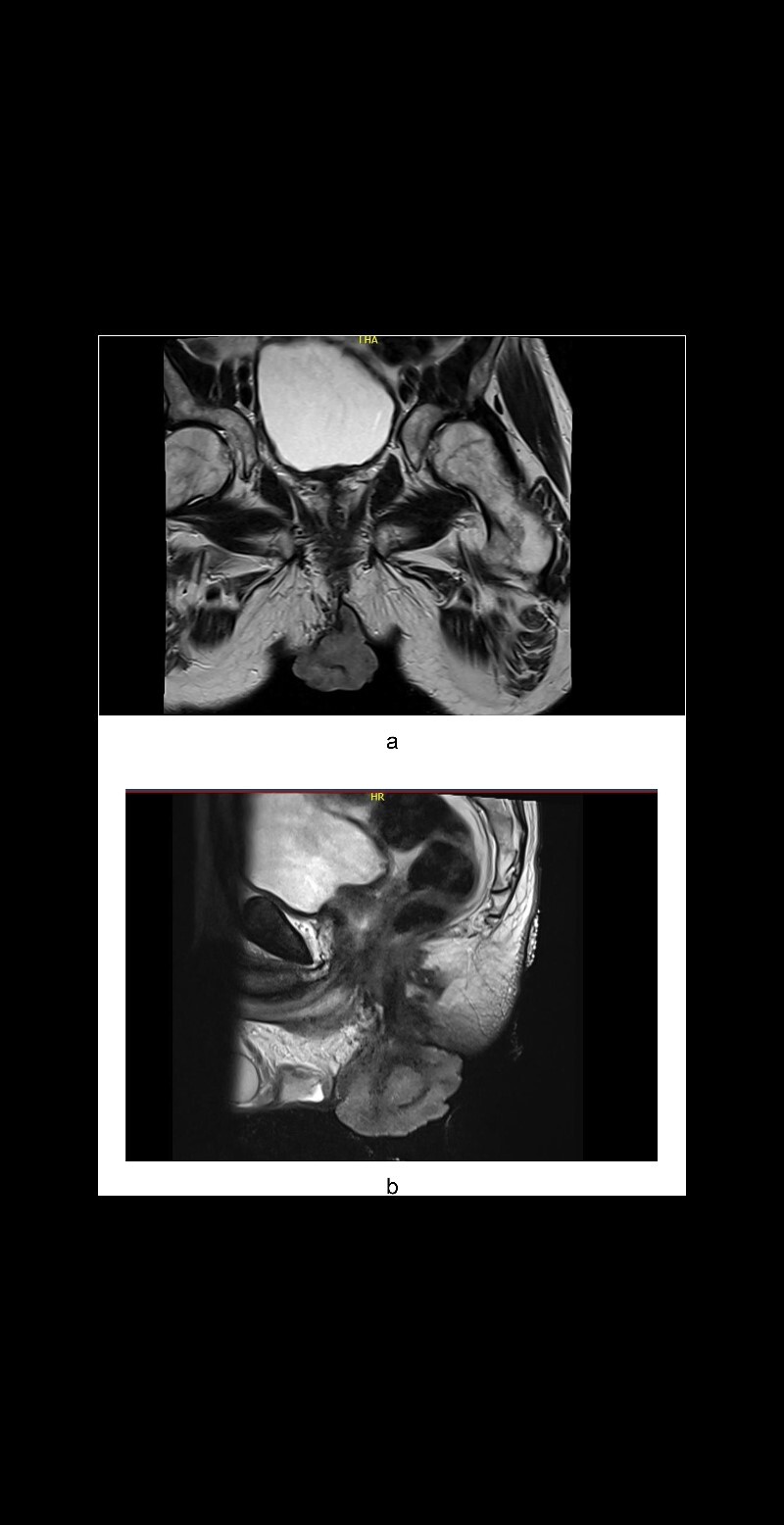
(a) Axial plane MRI pelvis: A large perianal mass. (b) Sagittal plane MRI pelvis: A large perianal mass

**Figure 2:**
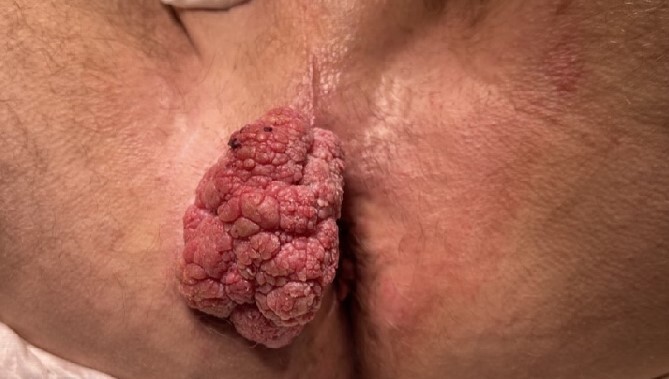
Perianal mass.

A male in his late 50s was referred to our surgical unit with a significant perianal mass, described as 10 cm × 15 cm, fungating, purulent, and malodorous, accompanied by fresh per rectum bleeding. Noted initially 12 months before presentation, the mass exhibited progressive enlargement. The patient's history of extensive smoking compounded the clinical concern. The patient reported no history of involvement in men who have sex with men behaviours. Diagnostic imaging, including CT of the abdomen and pelvis, showed no evidence of metastatic disease, and comprehensive virological screening was negative. Magnetic resonance imaging (MRI) of the rectum and pelvis (Fig. 1a and b) delineated a large perianal mass with characteristics suggestive of giant condylomata acuminata of Bushke–Löwenstein (Fig. 2), prompting further diagnostic refinement through examination under anaesthesia and targeted biopsies, which corroborated the suspicion.

This case stimulated comprehensive discussion at a colorectal multidisciplinary team meeting, leading to a consensus on the necessity for surgical resection of the mass, accompanied by consideration for V-Y flap reconstruction. Surprisingly, post-operative histological analysis unveiled a well to moderately differentiated invasive SCC, extending to 70 mm in diameter, with a pathological staging of pT3. This revelation underscored the malignant potential beneath the deceptive presentation, highlighting the surgical challenge in accurate pre-operative staging and the critical importance of histopathology in guiding therapeutic strategies.

Further histopathological examination confronted us with the complexities of accurately assessing the lesion's extent. The invasion depth was indeterminable due to the absence of adjacent normal epidermis, with the tumor thickness measured up to 42 mm. Extensive cautery artifact at the deep margin presented additional classification challenges, suggesting infiltrative SCC presence, thus reaffirming the pT3 staging.

In response to these findings, a comprehensive evaluation by the plastic surgery team recommended an advanced surgical strategy involving further wide excision paired with a flap-based reconstruction to address the defect and mitigate recurrence risks. The chosen inferior gluteal artery perforator (IGAP) flap reconstruction to the anal verge was meticulously planned to balance oncologic clearance with functional and aesthetic restoration. Post-operative analysis of the excised margins confirmed successful malignant tissue removal, evidenced by skin with dermal scarring and fibrosis indicative of previous surgical intervention, but importantly, devoid of residual invasive carcinoma.

This case illustrates the indispensable role of a multidisciplinary surgical approach in managing perianal masses, particularly those with hidden malignancies. The seamless integration of colorectal surgery, pathology, and reconstructive plastic surgery underscored our capacity to navigate complex diagnostic puzzles and achieve optimal patient outcomes through tailored, innovative surgical interventions.

## Discussion

This case underscores the inherent complexities in diagnosing and managing perianal masses, particularly when they masquerade as benign conditions. The patient's presentation with a large, symptomatic perianal mass initially suggested a diagnosis of giant condyloma acuminatum, a benign lesion, which upon surgical intervention, revealed an SCC. This diagnostic surprise highlights a critical aspect of perianal mass management: the reliance on histopathological examination for definitive diagnosis [1]. The association of HPV with anogenital malignancies, notably anal carcinoma and SCC, underscores a shared etiological risk factor, highlighting the necessity for vigilant HPV screening and vaccination in at-risk populations [1].

The surgical strategy employed in this case, involving excision followed by IGAP flap reconstruction, exemplifies the nuanced decision-making process in surgical oncology [5]. The transition from a presumed benign entity to a malignancy necessitated a reconsideration of the surgical approach to ensure both oncological clearance and functional preservation. The successful use of an IGAP flap for reconstruction post-wide excision showcases the advancements in surgical techniques that allow for effective defect management while minimizing the risk of recurrence [5, 6].

The challenge in preoperative staging, exacerbated by the extensive cautery artifact and the inability to assess the depth of invasion accurately, emphasizes the importance of intraoperative vigilance and the need for adaptable surgical planning [7]. The pathological finding of a well to moderately differentiated SCC with a pT3 staging, despite a non-indicative clinical and radiological profile, serves as a potent reminder of the unpredictable nature of perianal lesions [8].

The multidisciplinary approach to management, involving colorectal surgery, pathology, and plastic surgery, was pivotal in achieving a favourable outcome. This collaborative effort not only facilitated a comprehensive assessment and treatment plan but also underscored the importance of integrating various specialties to address complex surgical challenges effectively [9]. In the management of these malignancies, chemoradiotherapy emerges as a cornerstone, offering a non-invasive yet effective treatment modality that can preserve organ function and mitigate the need for extensive surgical interventions. This dual approach emphasizes the evolving landscape of treatment strategies, where integrating chemoradiotherapy with surgical expertise can optimize patient outcomes, balancing oncologic control with quality of life [10].

This case contributes to the existing literature by illustrating the potential for malignant transformation in perianal masses initially diagnosed as benign and underscores the necessity for a high degree of suspicion in their evaluation and management [10]. It reaffirms the role of surgical intervention in both the diagnosis and treatment of perianal masses, highlighting the critical need for histopathological confirmation to guide surgical decision-making [11]. Furthermore, it demonstrates the effectiveness of flap-based reconstruction techniques in managing significant perianal defects post-excision, offering valuable insights into achieving optimal oncological and functional outcomes [12, 13].

## Conclusion

The management of perianal masses remains a significant surgical challenge, necessitating a high index of suspicion for malignancy even in lesions initially presumed benign [13]. This case exemplifies the importance of a multidisciplinary approach, emphasizing the central role of surgery in the diagnostic pathway and the potential for innovative reconstructive techniques to enhance patient outcomes [14]. As surgical practices evolve, the continued sharing of such complex cases will be instrumental in refining our strategies for the effective management of perianal malignancies.
